# Moderate-to-good acceptability of smartwatch monitoring in head and neck cancer survivors: findings from the MOVE-1 feasibility study

**DOI:** 10.3389/fonc.2026.1844730

**Published:** 2026-06-03

**Authors:** Karl Vietinghoff, Susanne Fischer, Sabina Ulbricht, Daniel Strueder, Theresa Momper, Christian Junghanss, Sabine Felser

**Affiliations:** 1 Department of Internal Medicine, Clinic for Hematology, Hemostaseology, Oncology, Stem Cell Therapy and Palliative Care, Rostock University Medical Center, Rostock, Germany; 2 Mittelstand-Digital Zentrum Rostock, Stabsstelle Ärztlicher Vorstand, Rostock University Medical Center, Rostock, Germany; 3 Institute for Community Medicine, Department SHIP­KEF, University Medicine Greifswald, Greifswald, Germany; 4 Comprehensive Cancer Center – Mecklenburg-Western Pomerania (CCC-MV), University Medicine Greifswald, Greifswald, Germany; 5 Department of Otorhinolaryngology, Head and Neck Surgery “Otto Koerner”, Rostock University Medical Center, Rostock, Germany

**Keywords:** adherence, digital health technologies, health-related quality of life (HRQoL), physical activity, usability, wearables

## Abstract

**Background:**

Physical activity is associated with improved clinical outcomes across the cancer continuum. However, adherence to recommended activity levels among cancer survivors remains low. Digital health technologies such as smartwatches may support patients and survivors in achieving sufficient daily activity through continuous monitoring and feedback. Patients with head and neck cancer (HNC) often experience persistent functional limitations, yet evidence regarding the utility of wearable-based monitoring in this population remains limited. Therefore, the MOVE-1 study evaluated the feasibility of smartwatch-based monitoring in HNC survivors.

**Methods:**

MOVE-1 was a cross-sectional study investigating smartwatch use in HNC survivors. Participants were instructed to wear a smartwatch continuously for seven days (24 hours per day, 168 hours in total). Heart rate and step count were recorded and visible to participants via the smartwatch display. Feasibility parameters evaluated included recruitment rate, adherence assessed by heart rate data availability, frequency of display use (4-point Likert scale) and usability (System Usability Scale, SUS). Demographic and clinical characteristics were collected. Screened individuals who declined participation were analyzed separately regarding age, sex and reasons for refusal.

**Results:**

The recruitment rate was 50%. There were no significant differences between participants and non-participants regarding sex or age. Common reasons for non-participation included lack of interest, sufficient self-reported physical activity, time constraints and low affinity for technology. Thirty-five HNC survivors were enrolled (median age 63 ± 6 years). Median smartwatch wearing time was 111 hours, out of a total of 168 hours (67%). Display functions were used “often” or “very often” by 60% of participants. Perceived usability was rated as good, with a mean SUS score of 74 (percentile rank 69, grade B). Reported dissatisfaction mainly concerned wristband handling, while three participants experienced difficulties operating the smartwatch. The median daily step count was 7,298 steps.

**Conclusion:**

Moderate-to-good adherence and good usability suggest that smartwatch-based monitoring of physical activity and vital parameters is feasible in HNC survivors, although alternative wristband designs may improve usability. The observed step counts indicate that included individuals were more physically active than average. These findings support future interventional studies using smartwatches to promote physical activity in this patient cohort.

## 1 Introduction

Regular physical activity is increasingly recognized as an important component of survivorship care (1). Higher levels of physical activity after a cancer diagnosis have been associated with improved physical functioning, reduced cancer-related fatigue, fewer psychological symptoms and better health-related quality of life (HRQoL) (2). Moreover, high activity levels and less sedentary time are linked to improved disease-free survival and reduced cancer-specific, and overall mortality (3, 4). Despite these benefits, a substantial proportion of patients with cancer and cancer survivors remain insufficiently physically active (5).

Digital health technologies, including wearables such as smartwatches and activity trackers, offer new opportunities to monitor and promote physical activity in clinical populations. These devices enable continuous monitoring of behavioral and physiological parameters while supporting self-monitoring and goal setting. Observational studies indicate that cancer survivors using wearable activity trackers demonstrate higher levels of moderate-to-vigorous physical activity and better adherence to recommended activity levels than non-users (6, 7). In addition, treatment plans involving physical activity that integrate wearable technologies have demonstrated beneficial effects on cardiorespiratory fitness after a cancer diagnosis (8). Current evidence in oncology is mostly based on a limited number of tumor entities, particularly breast cancer (9), and often involves highly selected survivorship cohorts (9, 10). Therefore, the ability to apply those findings to other tumor types or larger cohorts is limited.

Head and neck cancers (HNC) represent a clinically vulnerable patient population characterized by substantial treatment-related morbidity and persistent functional impairments that may adversely affect long-term HRQoL (11–13). Nevertheless, the feasibility and acceptability of wearable-based monitoring in HNC patients and survivors remain poorly understood, particularly outside of North American settings (14). To address this gap, the MOVE-1 study aimed to replicate previous findings by evaluating the use of smartwatches to monitor physical activity and vital parameters in HNC in Mecklenburg–Western Pomerania, a federal state with the highest HNC incidence in Germany. Four categories were evaluated: (1) the proportion of HNC survivors ready to wear the smartwatch, (2) adherence to the wearing protocol, (3) smartwatch display use and (4) usability. Participants and researchers also reported advantages and disadvantages of the device, and furthermore, associations between physical activity and sociodemographics, behavioral factors and clinical characteristics were analyzed.

## 2 Methods

### 2.1 Study design

MOVE-1 was a single-center feasibility study with a cross-sectional design.

### 2.2 Study setting and participants

The study was conducted by the Clinic for Hematology, Hemostaseology, Oncology, Stem Cell Therapy, and Palliative Medicine in cooperation with the Department of Otorhinolaryngology, Head and Neck Surgery “Otto Koerner” (ENT-HNS) at Rostock University Medical Center (UMR). Eligible individuals were ≥ 18 years old with a confirmed diagnosis of HNC (ICD-10 C00–C14, C30–C32), were either undergoing antineoplastic treatment, palliative treatment or follow-up care, and had sufficient German language proficiency. Individuals not meeting these criteria or unable to provide informed consent were excluded. The study was conducted in accordance with the Declaration of Helsinki (15) and approved by the Ethics Committee of the University of Rostock (A2023-0149). It is registered at the German Clinical Trials Register (DRKS00032658).

### 2.3 Study procedure

The study procedure is shown in **Supplementary Figure 1A**. Potential participants were addressed and screened by the study team either during self-help group meetings or outpatient consultations at the ENT–HNS department. Potential participants from the ENT–HNS department were approached consecutively based on scheduled appointments, without preselection regarding mobility, physical functioning or affinity for technology. In addition, participants were offered flexible return options for the smartwatch, including home pick-up by the study team, to facilitate participation independent of mobility or travel constraints. Individuals not willing to participate were asked for two demographic information points (sex and age), if they engaged in private use of a smartwatch and their reasons for refusal. Written informed consent was obtained from those who had agreed to participate prior to them completing a baseline web-based questionnaire. Participants were instructed to wear the smartwatch for 24 hours a day over the following seven days. In addition to the device, they received a charging cable and a user manual, as well as a display usage log which they completed. Approximately 24 hours into the study, participants were contacted to ensure proper device functioning and to address potential questions. Upon device return and log submission, participants completed a follow-up web-based questionnaire. Participants received an incentive of €25, which could alternatively be donated to a charitable organization. Disease-specific clinical data at baseline were collected via a web-based questionnaire completed by the treating physician.

### 2.4 Data collection

The data collection schedule is summarized in **Table 1**. Treating physicians recorded tumor location, date of initial diagnosis, recurrence or second/third tumor, previous treatments, current therapy phase and, the date of completion of medical treatment. Tumor staging followed the Union for International Cancer Control (UICC) TNM classification, and human papillomavirus (HPV) status was determined by p16 immunohistochemistry.

**Table 1:** Data collection schedule.

Assessments	Baseline questionnaire	7-day wear period for the smartwatch	Follow up questionnaire	Data from the smartwatch and phone
Physician				
Disease-specific data Tumor location, date of diagnosis, recurrence or second/third tumor, UICC cancer stage, HPV p16 status, previous medical treatments, current therapy phase, date of completion of medical treatment	✓			
Participants				
Sociodemographic data and lifestyle factors Sex, age, height, weight, partnership, educational level, employment, zip code, household income, tobacco and alcohol consumption, level of physical activity (SGPALS) use of a smartwatch	✓			
Smartwatch display usage		daily		
Technical affinity (ATI) and experience with the smartwatch (SUS) Problems encountered and suggestions			✓	
EORTC QLQ-C30, H&N43			✓	
Smartwatch display usage total wearing period Recording of tracked data (e. g. step count)			✓	
Non-participants				
Reasons for refusal	✓			
Information Sex, age, use of a smartwatch	✓			
Study team				
Physical activity data (third-party applications Health Export – csv files) Step count, active calories, exercise minutes, stand hours				✓
Vital data (third-party applications Heart Reports – csv files) Heart rate				✓

UICC, Union for International Cancer Control; HPV, human papillomavirus; ATI, Affinity for Technology Interaction Scale; SUS, System usability Scale; EORTC QLQ-C30, Quality of Life questionnaire of cancer patients of European Organization for Research and Treatment of Cancer; H&N43, Quality of Life questionnaire module of head and neck cancer patients; SGPALS, Saltin-Grimby Physical Activity Level Scale.

The baseline questionnaire captured sociodemographics (sex, age, partnership (yes, no), educational level (≤ 10 and > 10 years), employment (yes, no), zip code (urban or rural area) and household income), anthropometric measures (height and weight) and behavioral aspects (tobacco and alcohol consumption and leisure-time physical activity (Saltin–Grimby Physical Activity Level Scale (16)) as well as smartwatch use. Body mass index was calculated and categorized according to standard WHO classifications (< 18.5, 18.5–24.9, 25.0–29.9 and ≥ 30.0, representing underweight, normal weight, overweight and obesity, respectively) (17).

Daily smartwatch display use was recorded using a 4-point Likert scale (“not at all” to “very often”). The follow-up questionnaire comprised the Affinity for Technology Interaction (ATI) Scale (18) and the System Usability Scale (SUS) (19). Participants were asked about advantages and disadvantages regarding smartwatch use. HRQoL was evaluated using the Quality of Life questionnaire of cancer patients of European Organization for Research and Treatment of Cancer (EORTC) QLQ -C30 (version 3.0) (20, 21) and the head-and-neck-specific QLQ-H&N43 (22). Overall display use during the wearing period was rated on a 4-point Likert scale and dichotomized into none/occasionally versus often/very often. Additionally, participants indicated which data they tracked in more detail (e.g., color-coded activity rings, step count, etc.).

### 2.5 Smartwatch devices and settings

Apple Watches were selected because, for security reasons, they were the only ones supported by the UMR’s IT department. In addition, considerations regarding technical support, device reliability, and product quality also contributed to the decision-making process. Seven Apple Watches (Apple Inc., Cupertino, CA, USA, Series 8 and 9, €550 to €600) paired with an iPhone SE (€530) were used according to institutional IT requirements. Six watches had a display size of 41 mm and one had a display size of 45 mm. Average battery life was approximately 18 hours. Devices were worn with the original Apple Sport strap in dark gray and operated in airplane mode, restricting functionality to basic features. The main screen was configured by the study team to allow all relevant data to be viewed at a glance. The Apple activity application displayed three colored rings representing daily goals: active energy expenditure (450 kcal/day), ≥ 30 minutes of physical activity and standing/moving ≥ 1 minute, in 12 hours/day (**Supplementary Figure 1B**). Additional parameters recorded included step count, active calories, distance covered and flights of stairs climbed. No individual customization of settings was performed. Fitness parameters and estimated energy expenditure based on heart rate data were recorded and could be viewed by the participants.

Participants were assigned a unique code for pseudonymization and a dedicated smartwatch prior to data collection. Participants’ age, height and weight were entered into the Apple Health app prior to the wearing period to ensure accurate data recording. Heart rate measurements were collected according to the device’s internal algorithm, which recorded intermittently. The recorded heart rate data were stored and processed entirely locally on the devices, without the availability of an internet connection. Activity data were extracted from Apple HealthKit using third-party applications (Heart Reports and Health Export) and exported as CSV files. Subsequently, the pseudonymized CSV files were securely transferred to the internal infrastructure for analysis.

### 2.6 Sample size calculation

This study was designed as a feasibility study; therefore, no formal sample size calculation based on statistical power was performed. A target sample size of 35 participants was defined *a priori* using a pragmatic approach, in line with methodological recommendations for pilot and feasibility studies (23).

### 2.7 Statistical analysis

Adherence was calculated as the proportion of smartwatch wearing time relative to the total study duration (24 hours x 7 days = 168 hours). Wearing time was inferred from heart rate recordings, with intervals of ≥ 30 minutes without heart rate data classified as non-wearing time (**Supplementary Figure 2**). This threshold was based on pilot testing conducted prior to the study and reflects expected recording patterns under daily-life conditions. During testing, fluctuations between heart rate recordings during normal wear generally occurred within the single-digit minute range. To account for variability in recording frequency and provide a conservative buffer, intervals of ≥ 30 minutes without heart rate recordings were classified as non-wearing time.

The SUS raw scores were standardized using a reference database and converted into percentile ranks, grades (A–F), adjective ratings and acceptance levels (24, 25) (**Supplementary Figure 3**).

Quantitative variables are presented as mean ± standard deviation or as median (Q1, Q3), ranging from minimum to maximum (min to max), whereas qualitative variables are presented as relative (%) and absolute (n) frequency of their occurrence. Missing data are indicated but not included in the calculation of percentage. The normal distribution of the data was checked using the Shapiro-Wilk test. No imputation of missing smartwatch-derived data was performed. Participants with missing data for a specific smartwatch-derived outcome across the entire monitoring period were excluded from the corresponding analysis.

Comparisons between groups (e.g., participants vs. non-participants) were conducted according to the level of measurement and data distribution. Categorical variables were compared using the chi-square test or Fisher’s exact test, while continuous variables were analyzed using the independent t-test or the Mann–Whitney U test, as appropriate.

Pearson or Spearman correlation coefficients were calculated to examine the strength and direction of relationships between quantitative variables (e.g., between adherence, display usage and SUS scores). Correlation coefficients r were interpreted as weak (0.10–0.29), moderate (0.30–0.49) or strong (≥ 0.50) (26). A *p*-value of < 0.05 was considered significant. All data were analyzed using IBM^®^ SPSS^®^ Statistics, Version 25 (IBM Corp., Armonk, NY, USA).

## 3 Results

### 3.1 Participation

**Figure 1:**
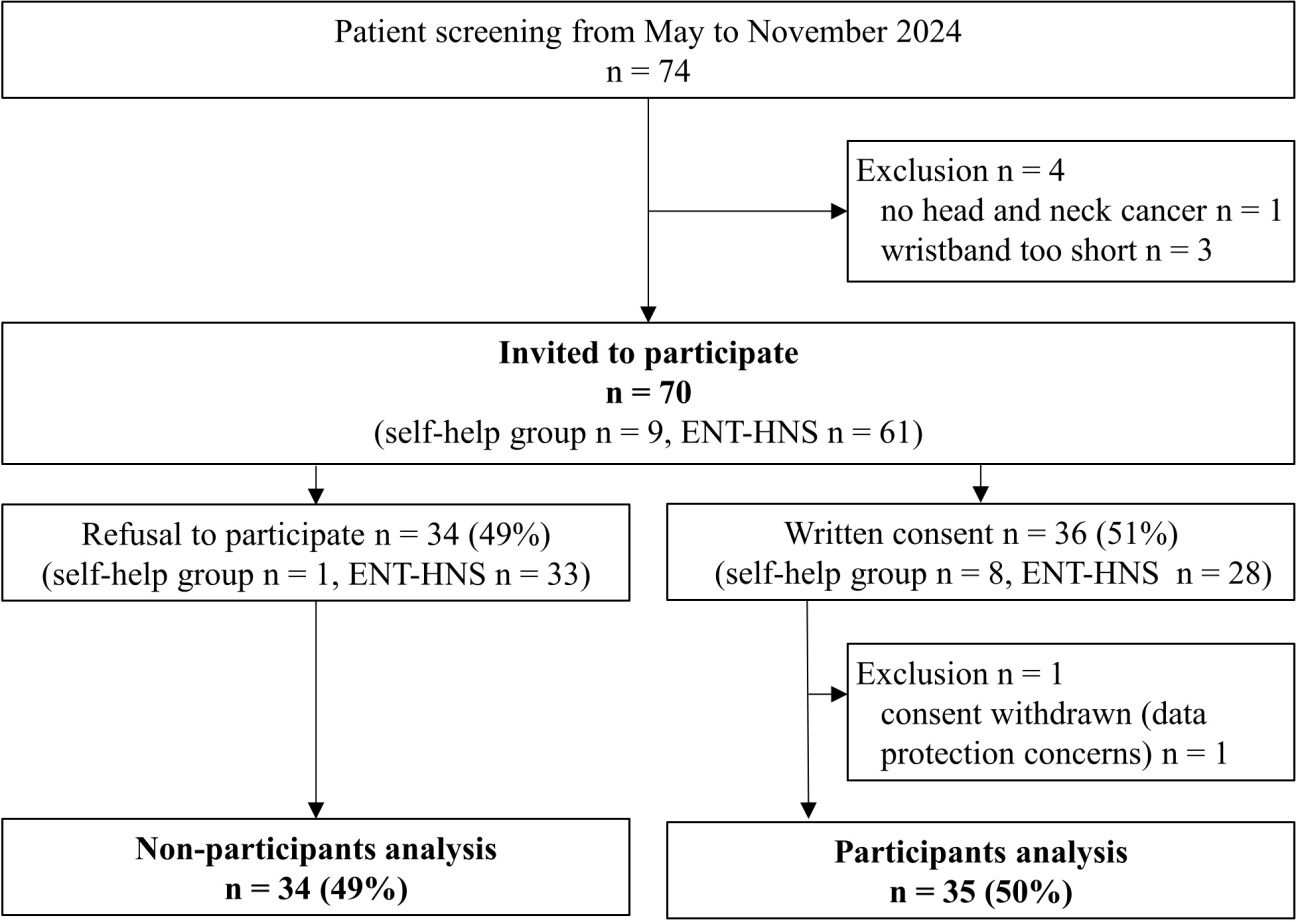
Flow chart MOVE-1 study. ENT-HNS, Department of Otorhinolaryngology, Head and Neck Surgery “Otto Koerner” at Rostock University Medical Center.

Between May and November 2024, 74 individuals were screened, of whom 70 met inclusion criteria. In the self-help group, 8 of 9 eligible persons (89%) consented, whereas 28 of 61 individuals (46%) in the ENT–HNS cohort participated. One participant withdrew consent due to concerns regarding data privacy, resulting in a final sample of 35 participants and a participation rate of 50% (**Figure 1**). Reported reasons for non-participation were lack of interest (n = 17, 40%), already being physically active (n = 7, 16%), low technical affinity (n = 6, 14%) and insufficient time (n = 6, 14%).

### 3.2 Sample characteristics

Participants (n = 35) were 54% male (n = 19), with a mean age of 63 ± 6 years (50 to 77), while non-participants (n = 34) were 76% (n = 26) male, mean age 66 ± 9 years (40 to 86). There were no significant differences in sex (*p* = 0.077) or age (*p* = 0.160). Most participants reported ≤ 10 years of schooling (n = 29, 83%) and 43% (n = 15) lived in rural areas. Proportions of smokers, ex-smokers, and non-smokers were 23% (n = 8), 69% (n = 24) and 8% (n = 3), respectively. Alcohol consumption of at least two times per week was reported by 40% (n =14), while 43% (n = 15) reported to abstain. Nearly all participants were physically active, with 57% (n = 20) exercising lightly (≥ 4 h/week) and 37% (n = 13) engaging in regular intensive activity (2–3 h/week). Private smartwatch use did not differ significantly between participants and non-participants (23% vs. 12%; *p* = 0.342). More details can be found in **Table 2**.

**Table 2:** Sociodemographic, lifestyle, and clinical data of participants and non-participants.

	Participants (n = 35)	Non-participants (n = 34)	*p*-value
Sociodemographic			
Sex, male	19 (54)	26 (76)	0.077
Age [years]	63 ± 6	66 ± 9	0.160
Body Mass Index [kg/m²]	24.7 ± 4.2		
<18.5	1 (3)		
18.5–24.9	18 (52)		
25.0–29.9	12 (34)		
≥30.0	4 (11)		
Living with a partner	28 (80)		
Secondary level of education	29 (83)		
Professional status, retired	25 (71)		
Residential area, rural area	15 (43)		
Net monthly household income [€] (n = 29)	1,950 (1,255, 3,400)		
Tobacco consumption			
Smoker	8 (23)		
Ex-Smoker	24 (69)		
Non-Smoker	3 (8)		
Current alcohol consumption			
None	15 (43)		
1–4 times/month	6 (17)		
>2 times/week	14 (40)		
Physical activity level			
Inactive	2 (6)		
Light (≥ 4 h/week)	20 (57)		
Regular exercise	13 (37)		
Smartwatch use	8 (23)	4 (12)	0.280
Tumor location			
Oropharynx	19 (54)		
Oral cavity	5 (14)		
Others	11 (32)		
UICC-cancer stage (n = 34)			
I + II	18 (53)		
III + IV	16 (47)		
Recurrence or second/third tumor	4 (11)		
HPV p16-status (n = 25), negative	13 (52)		
Time after initial diagnosis [months]	29 (17, 52)		
Treatment (n = 33)			
Surgery only	8 (24)		
RT or RCT only	11 (33)		
Surgery and RT/RCT	14 (43)		
Current therapy situation, complete remission	35 (100)^1^		

Data are presented as the number of participants (%) for categorical variables and as median (Q1, Q3) or as mean ± standard deviation.

UICC, Union for International Cancer Control; HPV, human papillomavirus; RT, radiotherapy; RCT, combined radio-chemotherapy.

^1^ For n = 31, the median time since the end of treatment was 26 (13, 49) months, with ranges from 0 to 160 months. One participant was still receiving immunotherapy but was in complete remission.

Clinically, the oropharynx was the most common tumor site (n = 19, 54%), followed by the oral cavity (n = 5, 14%). Almost half (n = 16, 47%) had UICC stage III–IV disease, and 11% (n = 4) had recurrent or multiple tumors. Among 25 tested individuals, 52% (n = 13) were HPV p16 negative. Median time since diagnosis was 29 (17, 52) months, and all participants were in complete remission. For n = 31, the median time since the end of treatment was 26 (13, 49) months, ranging from 0 to 160 months. Six participants (17%) were classified as long-term survivors (> 5 years after completion of treatment). One participant was still receiving immunotherapy but was in complete remission (**Table 2**).

### 3.3 HRQoL

Global HRQoL had a median score of 67 (42, 83), with no significant sex differences. Men reported higher physical function (93 vs. 80, *p* = 0.092), while women scored significantly lower on role, emotional and social functioning (*p* < 0.05). Predominant symptoms were dry mouth and thick saliva (50 (33, 83)), with women experiencing higher overall symptom burden, including pain, insomnia, anxiety and limited mouth opening (**Supplementary Table 1**).

### 3.4 Adherence to the wearing scheme

Median smartwatch wearing time was 111 hours (67% (57%, 88%) of a total of 168 hours; 39% to 97%). Devices were primarily removed for charging or showering. Heart rate data were recorded in approximately 240 second intervals (1,784 (1,343, 1,996) times per participant). One participant had missing device data for unknown reasons.

### 3.5 Display usage

After the wearing period, 60% (n = 21) of participants reported using the display often or very often. The colored activity rings were most frequently viewed (n = 32, 91%), followed by physical activity levels (n = 23, 66%) and step counts (n = 20, 57%). Display use declined from day 1 (n = 21, 60%) to day 7 (n = 15, 43%) (**Supplementary Figure 4**).

### 3.6 Affinity for technology

The mean ATI score was 3.7 ± 1.1 (1.3 to 6.0) and was moderately negatively correlated with age (r = –0.371, *p* = 0.028). Higher ATI scores were associated with prior smartwatch experience (4.3 ± 1.3 vs. 3.5 ± 1.0, *p* = 0.056), with a trend toward lower scores in rural participants (*p* = 0.060). No sex differences were observed.

### 3.7 Usability

The smartwatches achieved a mean SUS score of 74 ± 19 (median = 78 (60, 90)), corresponding to the 70th percentile (grade B), indicating good usability. Women reported higher SUS scores than men (86 (68, 95) vs. 73 (58, 80), *p* = 0.031). SUS scores were not associated with age or prior smartwatch experience. Seven participants (20%) reported device-related problems, most frequently difficulties putting on the watch (n = 4), wearing it (n = 2) or interpreting graphics (n = 2). Suggested improvements included changing the wristband (n = 8), a larger display (n = 3) and a longer battery life (n = 2).

### 3.8 Monitored physical activity

Median daily step count was 7,298 (4,883, 11,459) ranging from 3,595 to 15,315. Non-smokers had significantly higher median step counts than smokers (8,778 vs. 5,869; *p* = 0.019). Median weekly activity duration was 127 (48, 218) minutes (12 to 754). Again, non-smokers indicated higher activity duration than smokers (median of 185 min vs. 50 min; *p* < 0.001). There were no significant associations between activity duration and sex, age, alcohol consumption, HPV status, time after the end of treatment, HRQoL, functional scales or symptoms. The largest difference was observed between UICC stages (I–II 9,251 vs. III–IV 6,449; *p* = 0.051), suggesting a potentially meaningful trend despite not reaching statistical significance.

### 3.9 Predictors of adherence and display usage

Adherence was not significantly associated with sociodemographic, clinical, activity-related or technology-affinity variables. Display use correlated moderately with ATI (r_S_ = 0.384, *p* = 0.023) and SUS scores (r_S_ = 0.351, *p* = 0.038).

### 3.10 Advantages and limitations of the smartwatch

From a research perspective, Apple Watches were highly reliable and easy to use. Limitations included intermittent heart rate recording, short battery life, high cost and limited transparency regarding data processing.

## 4 Discussion

**Figure 2:**
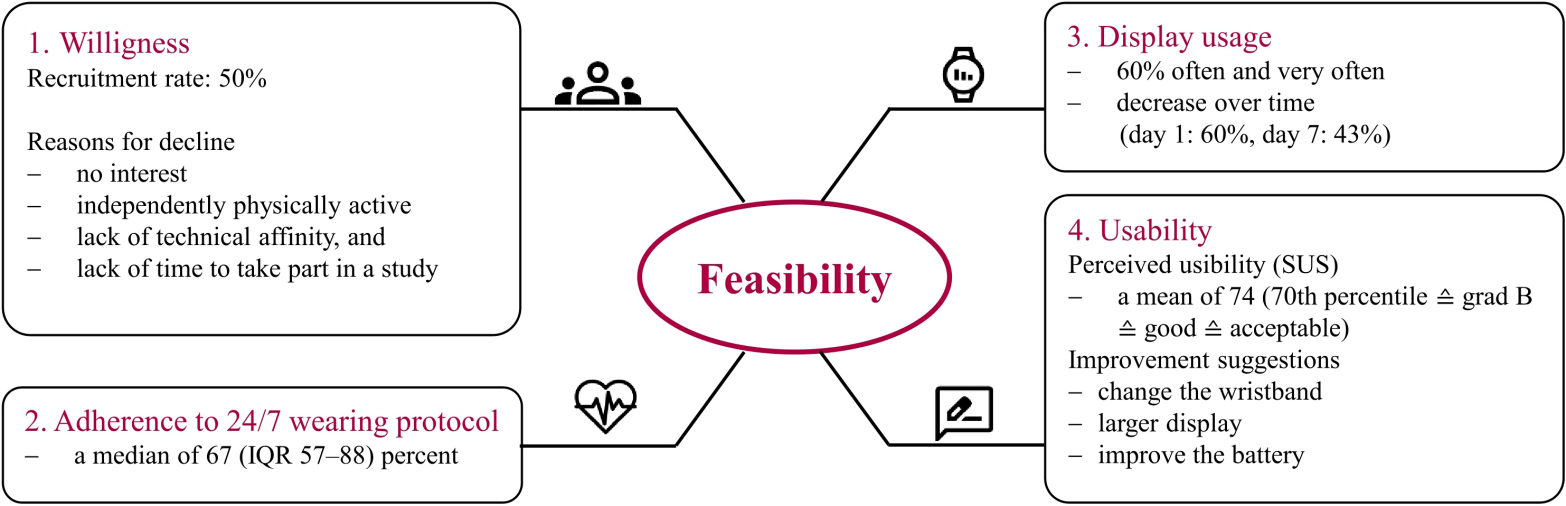
Criteria for assessing feasibility and results of the MOVE-1 study. IQR, inter quartile range; SUS, System Usability Scale.

Three main findings can be derived from this study. First, the recruitment of HNC survivors and the use of smartwatches in this population proved to be feasible, as demonstrated by a recruitment rate of 50%, a median adherence of 67% over the wearing period and moderate display use in 60% of participants (**Figure 2**). Second, from the participants’ perspective, the usability of the Apple Watch Series 8 and 9 was rated as good, with a mean score of 74 on the SUS, although limitations in usage remained. Third, the results support a distinction between adherence to the wearing scheme and use of the display. These two measures were not significantly correlated.

The recruitment rate of 50% (44% excluding self-help group participants) lies at the upper range compared to several studies involving cancer patients (median recruitment rate 38%) and is comparable to the median recruitment rates reported for survivors of prostate cancer (46%) and breast cancer (44%) (27). A similar study in patients with HNC reported a recruitment rate of 28% (28). The recruitment rate in our study may be explained by the short wearing period of the smartwatch as well as by strategies used to minimize barriers, such as integrating recruitment into clinical follow-up visits and offering to pick up the smartwatch at patients’ homes (29). Reasons for declining to participate in the study, such as lack of interest, limited time and low affinity for technology, are in line with other studies (30–33).

Adherence is typically defined as the proportion of time a device is worn (34). However, commercial smartwatches do not provide direct measures of wear time, requiring an indirect. Many studies therefore apply threshold-based approaches, such as defining a minimum number of daily steps to classify a valid wear day (35). These methods are heterogeneous and prone to misclassification, for example, when sporadic measurements are recorded despite the device not being worn (36), which limits comparability across wearable studies (14, 34, 37, 38). In the MOVE-1 study, adherence was estimated using a heart rate–based approach, in which gaps of ≥ 30 minutes in heart rate recordings were interpreted as non-wear periods. This method provides higher temporal resolution and reduces misclassification due to isolated measurements or brief device movements. However, the Apple Watch records heart rate at motion-dependent intervals rather than continuously (39); meaning that gaps may also occur during actual wear and thus potentially underestimate wear time. In addition, the relatively short battery life likely influenced adherence, as daily charging led many participants to remove the device overnight. Against this background, the observed median adherence of 67% can be considered moderate-to-good. For comparison, adherence rates between 31% and 92% have been reported in other HNC studies (14). Beyond rule-based approaches, data-driven methods may further improve the estimation of wear time in future studies. Machine learning models have already been applied to reconstruct wear patterns from wearable data (36), and large language models may also help distinguish between technically induced measurement gaps and actual non-wear periods.

While adherence describes device wear, display use reflects interaction with the technology. In the MOVE-1 study, both aspects were assessed, with 60% of participants reporting frequent or very frequent use of the display when starting to wear the watch. The decline in display use over time may be explained by the reduction of novelty which was also shown in other studies (40–42).

The usability of the Apple Watch devices used in this study was assessed using the SUS and reached a score of 74, indicating good usability (19). This exceeds the commonly reported benchmark value of 68 (43). Both usability and affinity for technology were significantly correlated with display use, suggesting that individual user characteristics play an important role in interactions with wearable devices. No technical problems occurred during the study period. The most frequently reported criticism concerned the wristband, particularly difficulties with fastening it. Some HNC survivors, especially those who had undergone reconstructive surgery using tissue grafts from the forearm, experienced discomfort while wearing the device. For future studies, selecting alternative bands with simpler fastening mechanisms, as well as offering variability in band length and color, may improve usability and acceptability. The exclusion of three potential participants due to insufficient wristband length further highlights the importance of providing different band sizes to reduce participation barriers. Despite the overall positive user ratings, several limitations exist from a research perspective when using the Apple Watch, including the limited transparency of proprietary measurement algorithms, restricted access to raw data export, short battery life and comparatively high acquisition costs.

Participants achieved a median of 7,298 steps per day. This level exceeds the average daily step count reported for the German general population (approximately 5,200 steps/day) (44) and the typically lower physical activity levels described in HNC patients and survivors (45–48). Previous studies have reported that cancer survivors accumulate approximately 4,660 to 11,000 steps per day, whereas patients undergoing active cancer treatment generally reach between 2,885 and 8,300 steps per day (9). The relatively high activity level observed in our cohort may partly reflect a healthy volunteer bias, as physically active individuals are more likely to participate in exercise-related studies (49). This interpretation is supported by the high physical function scores in the EORTC QLQ-C30, particularly among men (men 93 vs. women 80), and by the fact that only two participants reported being physically inactive. In addition, one quarter of participants were already using smartwatches prior to the study. Behavioral changes associated with wearing an activity monitor itself (Hawthorne effect) may also have temporarily increased physical activity levels (50). Although the smartwatch was not specifically used to increase activity in this feasibility study, participants received real-time feedback via the display. Our findings further suggest that HNC survivors with more advanced tumors and smokers tend to be less physically active, indicating that these groups may particularly benefit from targeted physical activity interventions. However, given the small sample size and exploratory nature of these analyses, these observations should be considered hypothesis-generating and require confirmation in larger studies.

### 4.1 Strengths and limitations

This feasibility study has several strengths and limitations. One methodological strength is the heart rate-based approach used to estimate device wear time, which identifies ≥ 30 minute gaps in heart rate recordings. This missing-value method allows a more temporally precise estimation of adherence than commonly used threshold-based approaches, and it reduces misclassification caused by sporadic measurements when the device is not worn. In addition, the study distinguished between adherence (device wear time) and active device use (display interaction), enabling a more differentiated assessment of engagement with the wearable technology.

However, some limitations should be considered. Epidemiological differences in HNC between Germany and regions such as North America and Scandinavia (51) may limit comparability with previous wearable studies, most of which were conducted in North American populations (14). In Germany, HPV-negative HNC is more prevalent, reflecting a higher proportion of lifestyle-associated tumors. Furthermore, within the MOVE-1 study, all participants were in complete remission, which may have introduced a selection bias by favoring functionally more stable individuals. In addition, the participants appeared to be more physically active than the broader HNC population, in which physical activity levels are generally low (45–48). Although several strategies were implemented to facilitate participation and reduce recruitment barriers, selection bias cannot be excluded. While limited mobility or low physical activity were not explicitly reported as reasons for declining participation, the study’s association with physical activity may nonetheless have discouraged less active individuals from participating. Moreover, the smartwatch provided real-time feedback (e.g., activity rings and step counts), which, in combination with the relatively short study duration and potential Hawthorne and social desirability effects, may have contributed to temporary behavioral changes and consequently increased step counts during the study period. In addition, functional status and frailty were not systematically assessed. The inclusion of measures such as ECOG performance status or simple functional assessments (e.g., handgrip strength or the 6-minute walk test) could have provided additional insights into participants’ physical condition and allowed for a more comprehensive evaluation of feasibility across different functional levels. Therefore, the study population may not be fully representative of the broader population of HNC patients and survivors, and the results may be less applicable to individuals undergoing active treatment, with lower baseline activity levels, greater frailty, greater functional limitations (e.g., due to a tracheostomy), or more complex clinical conditions.

The cohort also included a higher proportion of women than typically observed in the epidemiologically male-dominated HNC population (52), which may be partly explained by higher survival rates among women and a generally higher willingness to participate in health-related studies (53).

Although the follow-up questionnaire included open-ended questions that allowed participants to provide additional comments, these responses were not collected or analyzed within a structured qualitative framework. Consequently, they may not fully capture the range and depth of participants’ experiences and perceived barriers, including both positive and negative aspects of study participation.

## 5 Conclusion

The findings of the MOVE-1 study suggest that smartwatches are generally accepted by HNC survivors and can be used for the objective monitoring of physical activity and vital parameters in this population. The heart rate-based approach applied in this study provides a promising method for estimating wear time, with higher temporal resolution compared to commonly used threshold-based methods. For future intervention studies with larger sample sizes, the use of more cost-effective smartwatches with continuous heart rate recording may further improve the precision of adherence estimation using heart rate-based methods. To enhance participation as well as long-term wear and use, future studies should also consider devices with simple usability as well as interchangeable wristbands varying in material, fastening mechanism, length and color. Finally, our findings suggest that survivors with advanced HNC and smokers may represent populations of interest for future targeted interventions aimed at increasing everyday physical activity. At the same time, an important challenge for future research will be how to effectively engage a greater number of inactive HNC patients and survivors, as that cohort may benefit most from such interventions but are often underrepresented in exercise-related studies.

## Data Availability

The datasets presented in this study can be found in online repositories. The names of the repository/repositories and accession number(s) can be found below: The raw data supporting the conclusions of this article will be made available by the authors without undue reservation. The generated and analyzed datasets are available in the NFDI4Health repository: https://ldh.mediz-rostock.imise.uni-leipzig.de/projects/17.
