# Supplementary material for: Moderate-to-good acceptability of smartwatch monitoring in head and neck cancer survivors: findings from the MOVE-1 feasibility study
Source: Front Oncol. 2026 Jun 3;16:1844730. doi: 10.3389/fonc.2026.1844730 (PMC13271956; doi:10.3389/fonc.2026.1844730)
Supplement: Supplementary file 1 [file DataSheet1.pdf]

Figure S1. Study procedure MOVE-1 (A), and display settings and wristband (B)

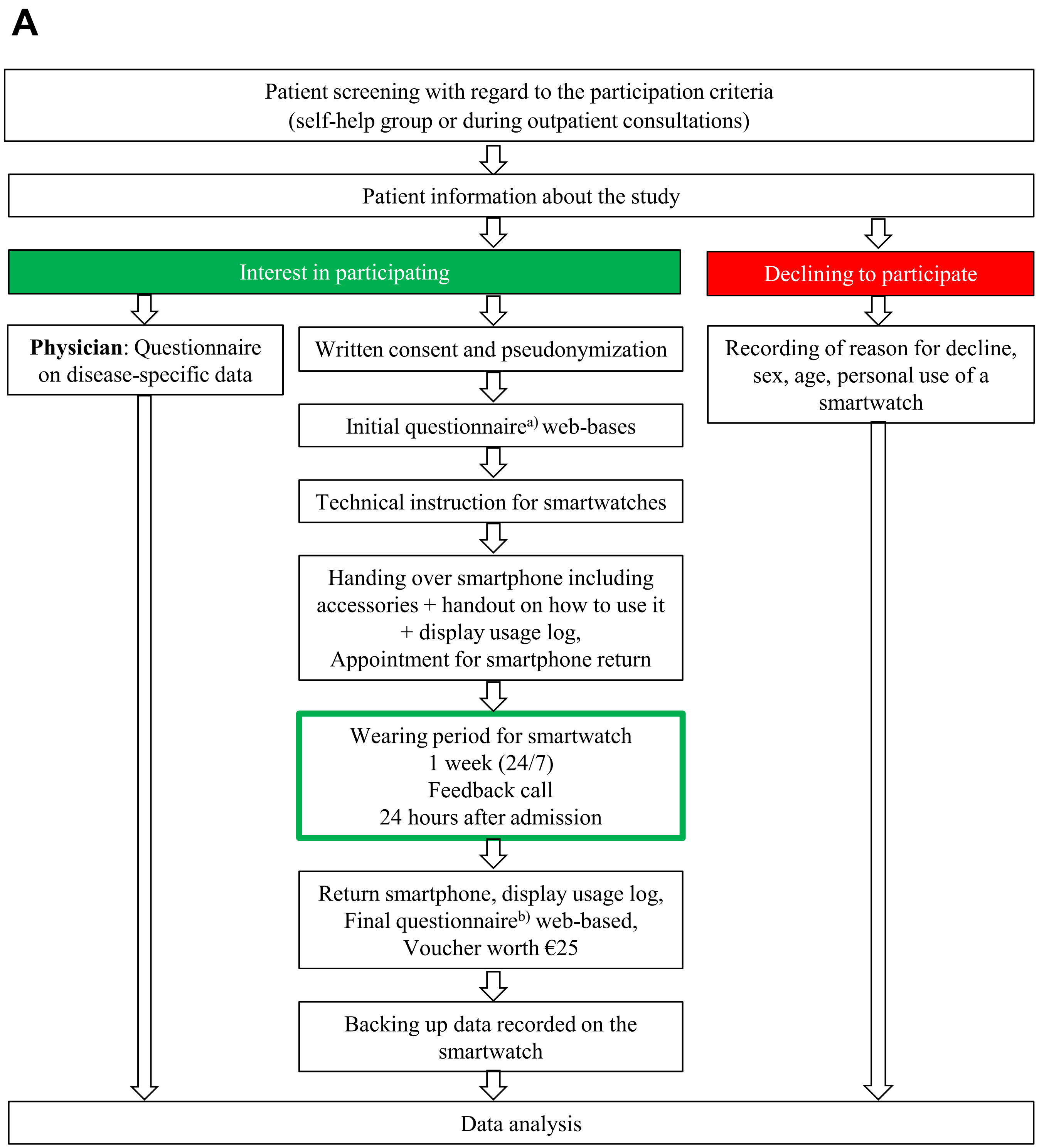

[a)] Sociodemographic data, lifestyle factors, Saltin-Grimby Physical Activity Level Scale, private use of a smartwatch
[b)] Affinity for Technology Interaction (ATI) Scale, System Usability Scale (SUS), problems using the smartwatch, EORTC-QLQ-C30 + Modul H&N43, frequency of display use during the entire wearing period

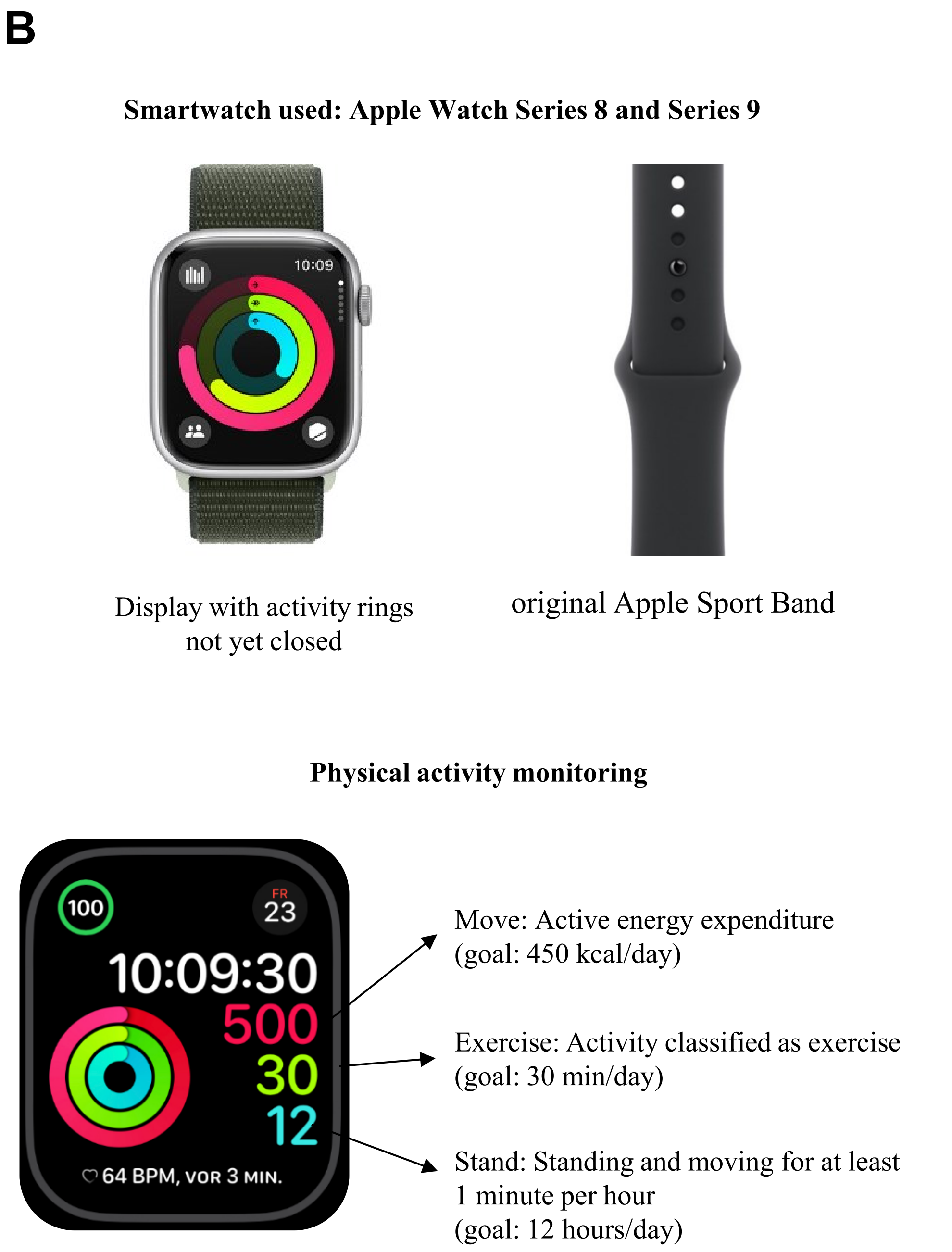

All participants used identical default settings. Additionally, the daily step count, active calories, number of stairs climbed, and distance traveled were recorded and could be tracked on the display.
