# Supplementary material for: Moderate-to-good acceptability of smartwatch monitoring in head and neck cancer survivors: findings from the MOVE-1 feasibility study
Source: Front Oncol. 2026 Jun 3;16:1844730. doi: 10.3389/fonc.2026.1844730 (PMC13271956; doi:10.3389/fonc.2026.1844730)
Supplement: Supplementary file 2 [file DataSheet2.pdf]

Figure S2. Calculation of adherence

1. Exporting raw data

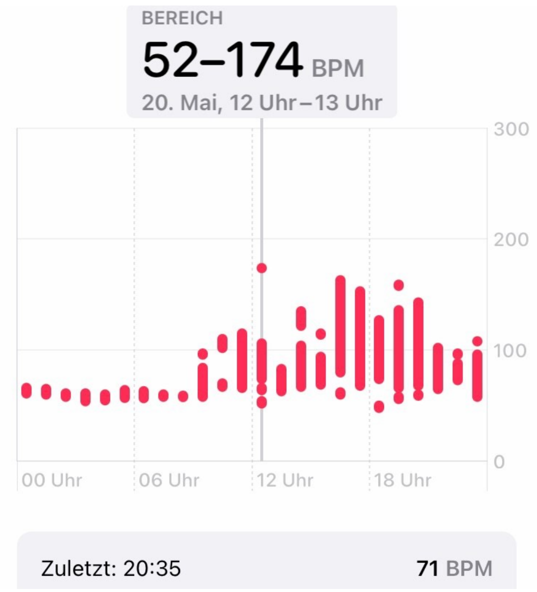

2. Calculation of time intervals

Date	Time	Heart rate	Time until next measurement
18.05.2024	12:35:33	84	00:01:17
18.05.2024	12:36:50	80	00:06:07
18.05.2024	12:42:57	83	00:39:00
18.05.2024	13:21:57	102	00:08:12
18.05.2024	13:30:09	80	00:01:17
18.05.2024	13:31:26	80	00:08:45
18.05.2024	13:40:11	84	00:02:43
18.05.2024	13:42:54	84	00:05:19
18.05.2024	13:48:13	85	00:07:48
18.05.2024	13:56:01	84	00:01:45
18.05.2024	13:57:46	80	00:04:26
18.05.2024	14:02:12	79	00:08:10
18.05.2024	14:10:22	73	00:12:37
18.05.2024	14:22:59	84	00:03:53
18.05.2024	14:26:52	73	00:02:16
18.05.2024	14:29:08	79	00:02:56
18.05.2024	14:32:04	73	00:30:29
18.05.2024	15:02:33	81	00:07:43
18.05.2024	15:10:16	77	00:01:15

red – time > 30 minutes

3. Calculation of adherence

(i) $$A_{n_x} = \frac{t_m - \sum t_{>AT}}{t_m}$$

(ii) $$A_{n_x} = \frac{168h - \sum t_{>0,5h}}{168h}$$

(iii) $$A_O = \frac{\sum A_{n_x}}{n}$$

A_{n_x} – adherence test person x
t_m – maximum total wear time
$t_{>AT}$ – time over the adherence threshold
A_O – overall adherence
n – number of test persons
