# Supplementary material for: Moderate-to-good acceptability of smartwatch monitoring in head and neck cancer survivors: findings from the MOVE-1 feasibility study
Source: Front Oncol. 2026 Jun 3;16:1844730. doi: 10.3389/fonc.2026.1844730 (PMC13271956; doi:10.3389/fonc.2026.1844730)
Supplement: Supplementary file 3 [file DataSheet3.pdf]

Figure S3. Interpretation of the System Usability Scale (SUS)

For a standardized and interpretable assessment of usability, the raw values of the System Usability Scale (SUS) were converted into several complementary interpretation formats using an extensive reference database with over 10,000 evaluations from more than 30 years. First, the SUS raw values were transformed into percentile ranks, which represent the relative position of a value within the reference distribution. The reference mean value is a SUS value of 68, which corresponds to the 50th percentile (Figure above).

In addition, the SUS values were categorized using a normalized grading system from A (very good usability) to F (unsatisfactory usability), with a grade of C representing average usability. This grading scale is consistent with the underlying normal distribution of the SUS values and the resulting percentile ranks.

For a verbal classification of usability, the SUS values were additionally linked to established adjective ratings. Based on the work of Bangor et al.*, numerical SUS values were associated with descriptive terms such as "excellent," "good," "satisfactory," and "poor." For example, SUS values above 85 correspond to usability perceived as "excellent," while values around 71 are rated as 'good' and values around 51 as "satisfactory."

In addition, the SUS values were classified into acceptance levels. Values above 70 were classified as "acceptable," values below 50 as "unacceptable." The range between 50 and 70 was defined as "barely acceptable." These acceptance categories correspond to the grade levels and percentile ranks and enable an additional, practical interpretation of the usability results.

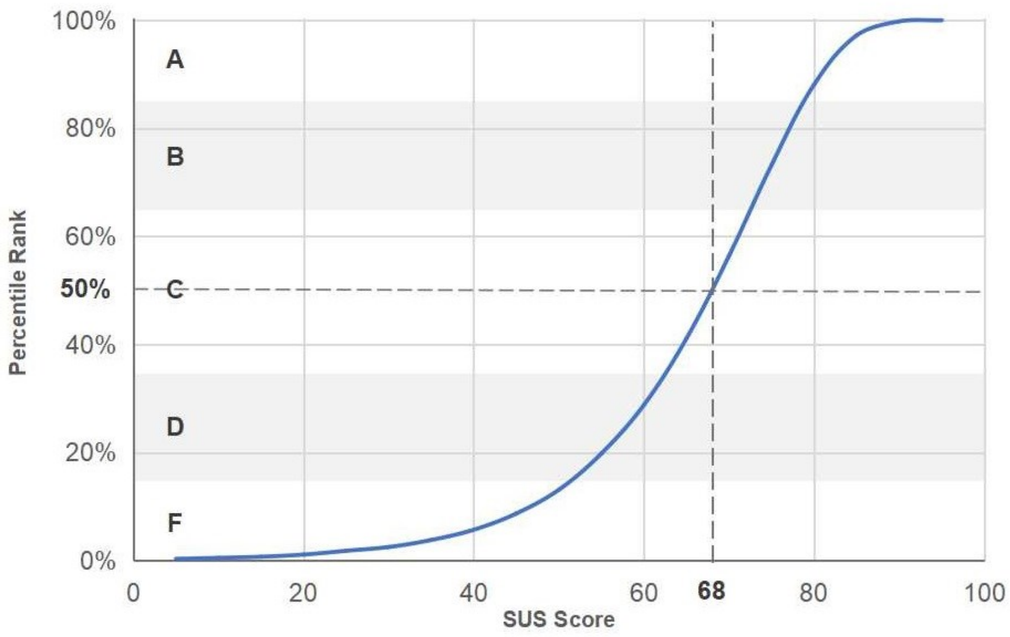

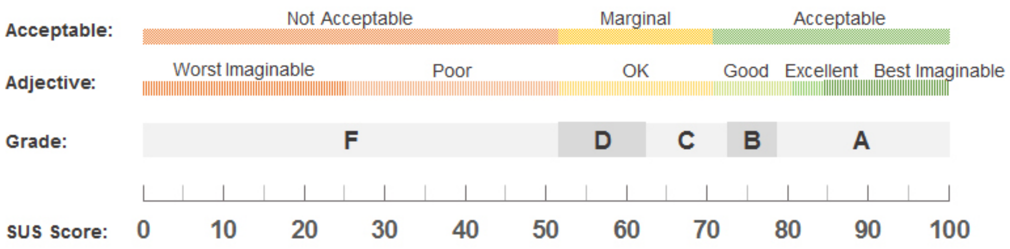

*Bangor, A et al. Determining What Individual SUS Scores Mean: Adding an Adjective Rating Scale. *Journal of usability studies*, 2009 (4),114-123.

Reference: https://measuringu.com/interpret-sus-score/
