# Supplementary material for: Moderate-to-good acceptability of smartwatch monitoring in head and neck cancer survivors: findings from the MOVE-1 feasibility study
Source: Front Oncol. 2026 Jun 3;16:1844730. doi: 10.3389/fonc.2026.1844730 (PMC13271956; doi:10.3389/fonc.2026.1844730)

Figure S4 Overall assessment of display usage over the one-week wear period (A) and change in display usage over time (B)

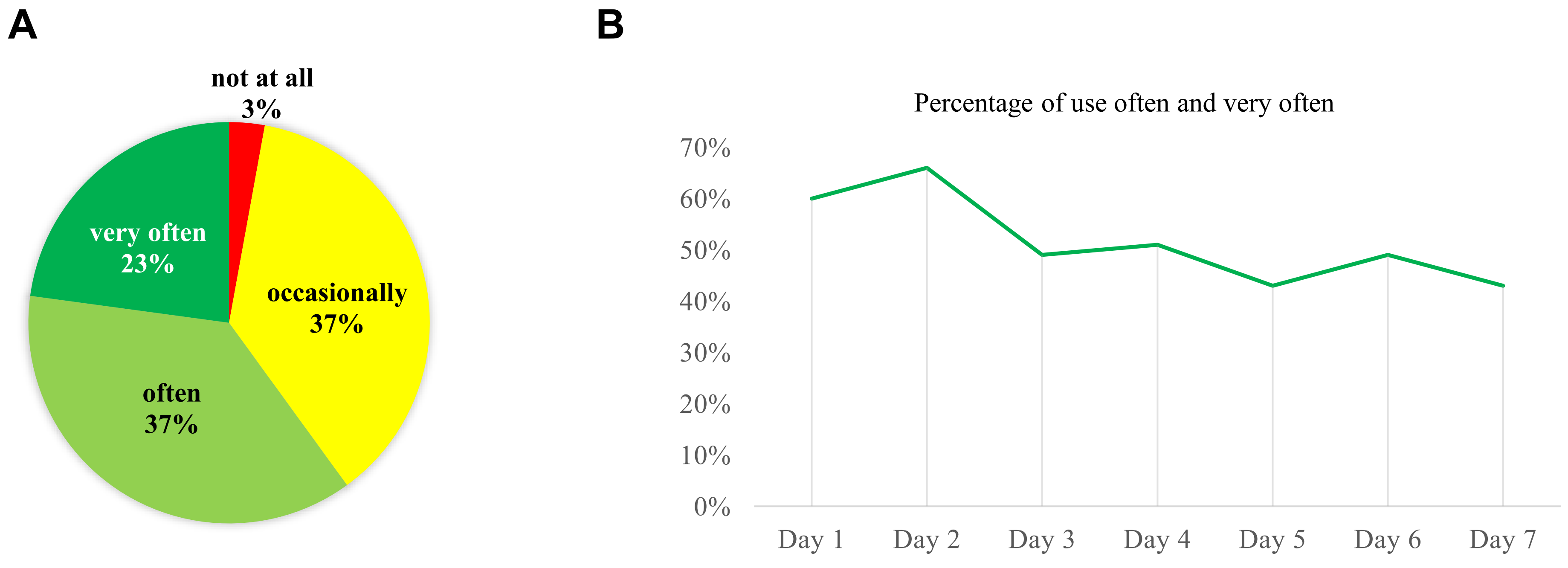

Supplement: Supplementary file 4 [file DataSheet4.pdf]
