# Supplementary material for: Moderate-to-good acceptability of smartwatch monitoring in head and neck cancer survivors: findings from the MOVE-1 feasibility study
Source: Front Oncol. 2026 Jun 3;16:1844730. doi: 10.3389/fonc.2026.1844730 (PMC13271956; doi:10.3389/fonc.2026.1844730)
Supplement: Supplementary file 5 [file Table1.docx]

**Table S1.** Quality of life and symptoms depending on sex

| **Parameter** | **Total cohort**  **(n = 35)** | **Men**  **(n = 19)** | **Women**  **(n = 16)** | ***p-*value** | |
| --- | --- | --- | --- | --- | --- |
| **QoL (EORTC QLQ-C30^a^)** |  |  |  |  |  |
| Global QoL scale [0-100] | 67 (42, 83) | 67 (42, 83) | 63 (42, 73) | 0.235 | |
| Functional scales [0-100]  physical  role  emotional  cognitive  social | 87 (67, 93)  67 (33,100)  75 (50, 92)  83 (67, 100)  67 (50, 100) | 93 (80, 100)  100 (67, 100)  83 (67, 100)  83 (67, 100)  100 (67, 100) | 80 (67, 87)  59 (33, 67)  58 (44, 81)  83 (67, 83)  59 (21, 83) | 0.092  0.007*  0.008*  0.583  0.040* | |
| Symptom scales [0-100]  fatigue  nausea/ vomiting  pain | 33 (11, 56)  0 (0, 0)  33 (0, 67) | 22 (0, 44)  0 (0, 0)  17 (0, 33) | 39 (25, 56)  0 (0, 0)  50 (4, 67) | 0.099  0.086  0.039* | |
| Single items [0-100]  dyspnea  insomnia  appetite loss  constipation  diarrhea  financial difficulties | 0 (0, 33)  33 (0, 67)  0 (0, 33)  0 (0, 33)  0 (0, 0)  0 (0, 33) | 0 (0, 33)  0 (0, 33)  0 (0, 33)  0 (0, 0)  0 (0, 0)  0 (0, 0) | 17 (0, 67)  33 (33, 67)  33 (0, 67)  0 (0, 33)  0 (0, 0)  0 (0, 58) | 0.333  0.016*  0.053  0.459  0.086  0.069 | |
| **QoL (EOTRC QLQ-HN43^b^)** |  |  |  |  | |
| Symptom scales [0-100]  pain in the mouth  swallowing  problems with the teeth  dry mouth and ticky salvia  problems with senses  speech  body image  social eating  sexuality  shoulder problems  skin problems  anxiety | 17 (0, 35)  17 (0, 50)  33 (0, 44)  50 (33, 83)  33 (0, 33)  13 (0, 47)  11 (0, 33)  17 (0, 42)  0 (0, 67)  17 (0, 67)  11 (0, 22)  33 (17, 67) | 17 (0, 25)  8 (0, 33)  11 (0, 33)  50 (33, 83)  17 (0, 33)  13 (0, 13)  11 (0, 22)  0 (0, 25)  0 (0, 33)  0 (0, 33)  0 (0, 17)  33 (0, 50) | 17 (0, 40)  33 (2, 56)  33 (11, 67)  50 (33, 83)  33 (0, 46)  23 (7, 65)  22 (3, 53)  29 (8, 65)  33 (0, 67)  50 (0, 67)  11 (0, 22)  59 (21, 96) | 0.756  0.145  0.085  0.613  0.756  0.051  0.056  0.015*  0.098  0.079  0.301  0.022* | |
| Symptom items [0-100]  opening mouth  coughing  social contact  swelling in the neck  weight loss  problems with wound healing  neurological problems | 33 (0, 67)  0 (0, 33)  0 (0, 0)  0 (0, 33)  0 (0, 33)  0 (0, 33)  33 (0, 67) | 0 (0, 33)  0 (0, 67)  0 (0, 0)  0 (0, 33)  0 (0, 33)  0 (0, 0)  33 (0, 33) | 67 (0, 100)  0 (0, 33)  0 (0, 33)  17 (0, 33)  0 (0, 33)  0 (0, 33)  33 (0, 67) | 0.011*  0.452  0.012*  0.795  1.000  0.127  0.404 | |

The Mann-Whitney-U-test (exact significance 2-tailed) was used to compare men and women scores.

* *p* >0.05

Abbreviations: QoL, Quality of Life

^a^Quality of Life questionnaire of cancer patients of European Organization for Research and Treatment of Cancer,

^b^Head and neck–specific questionnaire of European Organization for Research and Treatment of Cancer,

A high value on the scale ‘global QoL’ and on the functional scales means a high degree of subjectively perceived health and a high assessment of the QoL or a high degree of performance and function. A high value in the symptom scales correlates with a high degree of complaints.
